# Ⅲa期非小细胞肺癌术后生存分析

**DOI:** 10.3779/j.issn.1009-3419.2013.11.06

**Published:** 2013-11-20

**Authors:** 慧慧 刘, 燕 徐, 孟昭 王, 克 胡, 满姣 马, 巍 钟, 力 张, 静 赵, 龙芸 李, 华竹 王

**Affiliations:** 1 100730 北京，中国医学科学院北京协和医院呼吸内科 Department of Respiratory Medicine, Peking Union Medical College Hospital, Beijing 100730, China; 2 100730 北京，中国医学科学院北京协和医院放疗科 Department of Radiotherapy, Peking Union Medical College Hospital, Beijing 100730, China

**Keywords:** 肺肿瘤, 化疗, 放疗, 影响因素, Lung neoplsms, Chemotherapy, Radiotherapy, Influencing factor

## Abstract

**背景与目的:**

Ⅲa期非小细胞肺癌（non-small cell lung cancer, NSCLC）目前多提倡手术治疗, 术后患者的生存情况则受多种因素的影响。该研究对Ⅲa期NSCLC手术患者的预后影响因素进行了统计分析。

**方法:**

回顾性分析2002年3月-2012年10月北京协和医院收治的术后病理确诊为Ⅲa期NSCLC的患者151例。按照N分期的不同分为T4N0/T3-4N1M0和T1-3N2M0期两组。采用*Kaplan-Meier*方法比较生存期（overall survival, OS）和无进展生存期（progression free survival, PFS）, 进行OS和PFS影响因素的单因素分析, 并绘制生存曲线。采用*Cox*比例风险模型进行多因素分析。以*P* < 0.05认为差异有统计学意义。

**结果:**

151例Ⅲa期NSCLC患者中有T4N0/T3-4N1M0期43例和T1-3N2M0期108例。全组患者的中位OS和PFS分别为38.9月和19.2月。T4N0/T3-4N1M0和T1-3N2M0期的中位OS分别为48.7月和38.9月, 中位PFS分别为14.9月和19.8月。两组OS和PFS的差异均无统计学意义（*P* > 0.05）。单因素和多因素分析均显示术后化疗对Ⅲa期手术患者OS的影响有统计学意义（*P*=0.001）, 肿瘤家族史对其PFS的影响有统计学意义（*P* < 0.05）。肿瘤最大径只在单因素分析中对PFS有影响。

**结论:**

术后化疗可以改善Ⅲa期NSCLC手术患者的生存期, 术后放疗对生存期无改善作用。

## Abstract (en)

**Background and objective:**

At present, surgery is advocated for stage Ⅲa non-small cell lung cancer (NSCLC), and the survival of them is determined by many factors. The aim of this study is to analyze the influencing factors of prognosis for stage Ⅲa surgical patients.

**Methods:**

Between March 2002 and October 2012, 151 surgical cases that have postoperative pathological finding of stage Ⅲa NSCLC with completed followed-up data were received in the Peking Union Medical College Hospital. According to different N stages, 151 patients were divided into T4N0/T3-4N1M0 and T1-3N2M0 stages. *Kaplan-Meier* survival method was used to calculate the overall survival (OS) and progression-free survival (PFS), and to proceed univariate analysis of survival. *Cox* regression analysis was used to conduct multivariate analysis. A p-value less than 0.05 was evaluated as statistically significant.

**Results:**

151 stage Ⅲa NSCLC patients had 43 stage T4N0/T3-4N1M0 cases and 108 stage T1-3N2M0 cases. The median OS and PFS of the whole group were 38.9 and 12.9 months respectively. The median OS of stage T4N0/T3-4N1M0 and T1-3N2M0 were 48.7 and 38.9 months. The median PFS of them were 14.9 and 19.8 months respectively. There were no significant differences of OS and PFS between two groups. Univariate and multivariate analysis indicated that postoperative chemotherapy had a significant influence on OS of the surgical patients with stage Ⅲa NSCLC (*P*=0.001), and family history of tumor had a significant influence on PFS (*P* < 0.05). The maximum diameter of tumor had a significant influence on PFS only in univariate analysis.

**Conclusion:**

For stage Ⅲa NSCLC, postoperative chemotherapy can increase OS and PFS, but postoperative radiotherapy have no benefit on them.

肺癌是目前世界范围内发病率和死亡率最高的恶性肿瘤，非小细胞肺癌（non-small cell lung cancer, NSCLC）占全部肺癌的80%。其中约1/3的患者在最初诊断时已处于局部晚期（Ⅲa期/Ⅲb期），对于可以手术切除的Ⅲa期NSCLC患者，手术仍是其治疗方式之一；也有部分患者术后病理才确定纵隔淋巴结转移。这些患者术后生存情况的影响因素越来越受到人们的关注。因此，我们对2002年3月-2012年10月在北京协和医院胸外科进行手术切除且病理确诊为Ⅲa期NSCLC的151例患者进行统计分析，为进一步判断其预后影响因素提供依据。

## 1 对象与方法

### 1.1 研究对象

2002年3月-2012年10月于北京协和医院就诊的NSCLC患者151例。所有患者均符合以下条件：①术前通过胸腹增强CT、全身骨ECT、头部增强MRI等检查排除远处转移；②接受了肺癌根治术（肺叶切除或全肺切除+纵隔淋巴结清扫术）；③术后病理为NSCLC，病理分期为Ⅲa期；④年龄18周岁以上；⑤体能状态ECOG评分0分-2分；⑥无严重的心、肝、肾和造血系统等疾病；⑦规律随访，病历资料完整。

### 1.2 研究方法

回顾性分析151例Ⅲa期NSCLC患者的病历资料。包括患者的性别、年龄、吸烟情况、ECOG评分、肿瘤史、肿瘤家族史、病理分型、分化程度、TNM分期、治疗方案、进展及生存情况。151例患者按照N分期的不同又可分为T4N0/T3-4N1M0期和T1-3N2M0期两组。在单因素分析中分析性别、年龄、吸烟情况、肿瘤史、肿瘤家族史、ECOG评分、病理分期（tumor-node-metastasis, TNM）、病理类型、分化程度、术后辅助化疗或放疗、手术方式、肿瘤最大径、纵隔淋巴结阳性数及隆突下淋巴结是否阳性对Ⅲa期NSCLC手术患者OS和PFS的影响；在多因素分析中分析性别、年龄、吸烟情况、肿瘤史、肿瘤家族史、ECOG评分、病理分期（TNM）、病理类型、分化程度、术后辅助化疗或放疗对Ⅲa期NSCLC手术患者OS和PFS的影响。以门诊或电话形式随访。随访时间至患者死亡或2013年4月15日截止，时间以月表示。

### 1.3 观察指标

总生存期（overall survival, OS）定义为患者从病理确诊日期开始至死亡或末次随诊的时间（月）。无进展生存期（progression-free survival, PFS）定义为患者从病理确诊日期开始至疾病进展或疾病尚未进展的末次随诊时间（月）。

### 1.4 统计学分析

统计学分析采用SPSS 18.0软件。利用*Kaplan-Meier*方法比较OS和PFS，进行生存期影响因素的单因素分析，并绘制生存曲线，生存曲线无交叉时采用*Log-rank*检验，有交叉时采用*Tarone-Ware*检验。采用*Cox*比例风险模型进行多因素分析。以*P* < 0.05认为差异有统计学意义。

## 2 结果

### 2.1 一般情况

151例Ⅲa期NSCLC手术患者的一般临床特征见表 1。

**1:** Ⅲa期各组患者的临床特征 Clinical characteristics of each group of stage Ⅲa

Characteristic		*n*	Ratio (%)
Gender			
	Male	111	73.5
	Female	40	26.5
Age (yr)			
	＜60	66	43.7
	≥60	85	56.3
Smoke			
	No	57	37.7
	Yes	94	62.3
History of tumor			
	No	139	92.1
	Yes	12	7.9
Family history of tumor			
	No	122	80.8
	Yes	29	19.2
Performance status			
	0	127	84.1
	1	24	15.9
Stage Ⅲa			
	T4N0/T3-4N1	43	28.5
	T1-3N2	108	71.5
Histology			
	Squamous	72	47.7
	Non-squamous	79	52.3
Differentiation			
	Poor	68	45.0
	Moderate	56	37.1
	High	16	10.6
	Unknown	11	7.3

### 2.2 治疗情况

151例患者的手术方式主要有两种，肺叶切除+纵隔淋巴结清扫术者138例，全肺切除+纵隔淋巴结清扫术者13例。术后辅助化疗者共102例，多采用铂类为主的联合化疗方案，除7例在外院化疗用药方案不详外，化疗方案明确的95例患者中采用铂类为主联合化疗方案的占95.8%（91/95）。联合化疗药包括吉西他滨（31例）、长春瑞滨（18例）、紫杉醇（18例）、多西紫杉醇（13例）、培美曲塞（5例）、VP-16（4例）、长春地辛+丝裂霉素（1例）和5-Fu（1例）。非铂类化疗方案包括1例单药力比泰、1例单药吉西他滨、1例VP-16+异环磷酰胺及1例紫杉醇+5-Fu。除6例化疗周期数不详外，其余96例患者共化疗410周期，平均4.3。术后放疗采用普通放疗或三维适形放疗（3D-CRT），照射野包括原发灶、同侧肺门及相应纵隔淋巴引流区，然后改前后斜野避开脊髓局部加量照射，部分行双侧锁骨上区补量照射。采用常规分割，1.8 Gy/次-2 Gy/次，1次/1天，5次/周。全组151例患者中有69例行术后放疗，11例放疗剂量不详，2例未按原计划完成放疗方案，其余56例放疗总剂量为2, 942.6 Gy，平均52.5 Gy。术后辅助化疗或放疗，一般于术后1个月-2个月开始进行，部分患者因身体状况较差延迟到术后3个月-4个月进行。

根据NCCN指南，术后病理为T4N0/T3-4N1M0期的Ⅲa期NSCLC患者标准治疗方式为术后辅助化疗，T1-3N2M0期的标准治疗方式为术后辅助化疗+放疗。本研究中Ⅲa期NSCLC手术患者的治疗情况见图 1。

**1:**
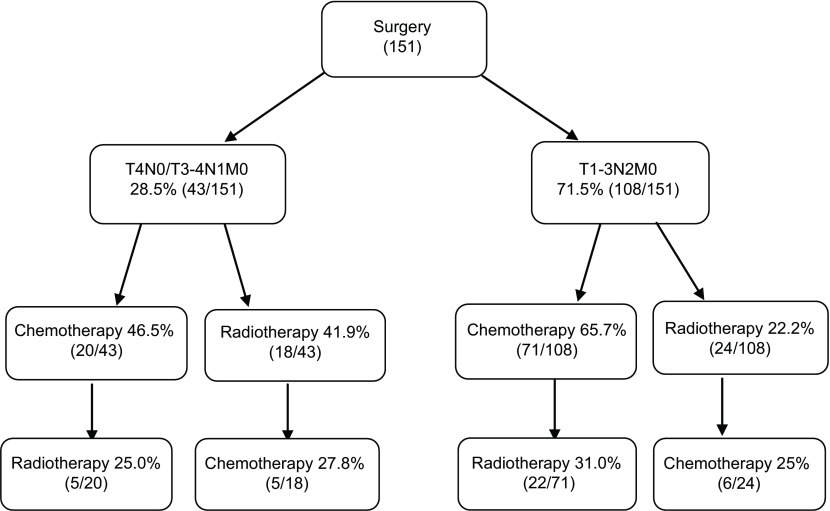
151例患者的治疗情况 The introduction of the treatment of 151 patients

### 2.3 全组的OS和PFS

全组151例Ⅲa期NSCLC手术患者的中位OS为38.9个月，中位PFS为19.2个月。Ⅲa期分T4N0/T3-4N1M0期43例和T1-3N2M0期108例两组。两组的中位OS分别为48.7个月和38.9个月，差异无统计学意义（χ^2^=0.010, *P*=0.922）；中位PFS分别为14.9个月和19.8个月，差异亦无统计学意义（χ^2^=0.023, *P*=0.880）。

### 2.4 OS和PFS影响因素分析

#### 2.4.1 OS

单因素分析显示只有术后辅助化疗对Ⅲa期NSCLC手术患者OS的影响有统计学意义（表 2）。多因素分析也显示只有术后辅助化疗使死亡风险降低了55.1%（*P*=0.001, HR=0.449, 95%CI: 0.276-0.731）。术后辅助化疗与未辅助化疗者的OS曲线见图 2。Ⅲa期中T1-3N2M0期的术后辅助放疗者有46例，未放疗者62例。两组的中位OS分别为30.4个月和40.7个月，其差异无统计学意义（χ^2^=2.414, *P*=0.120）。

**2:** 151例患者总生存的单因素分析 Univariate analysis of overall survival (OS) of 151 patients

Factor		OS/month	95%CI	*χ*^2^	*P*
Gender				0.487	0.485
	Male	36.1	19.16-53.04		
	Female	42.7	28.38-57.08		
Age（yr）				1.847	0.174
	< 60	44.0	29.20-58.87		
	≥60	31.6	18.72-44.48		
Smoke				0.751	0.386
	No	40.7	28.93-52.41		
	Yes	31.6	14.68-48.52		
History of tumor					
	No	36.1	24.01-48.19		
	Yes	65.9	0.00-135.42		
Family history of tumor				0.134	0.714
	No	38.9	21.77-56.09		
	Yes	44.0	21.95-66.11		
Performance status				2.639	0.104
	0	44.0	27.48-60.58		
	1	24.7	22.00-27.40		
Stage Ⅲa				0.010	0.922
	T4N0/T3-4N1	48.7	12.62-84.72		
	T1-3N2	38.9	26.74-51.12		
Histology				3.651	0.056
	Squamous	31.6	21.73-41.47		
	Non-squamous	44.0	25.81-62.25		
Differentiation				4.587	0.205
	Poor	31.6	17.99-45.21		
	Moderate	30.3	13.47-47.19		
	High	58.4	25.96-90.90		
	Unknown	-	-		
Adjuvant chemotherapy				10.920	0.001
	No	23.0	19.15-26.91		
	Yes	44.0	31.60-56.46		
Adjuvant radiotherapy				0.747	0.387
	No	36.1	22.37-49.83		
	Yes	38.9	20.85-57.01		
Style of surgery^#^				2.213	0.137
	Lobectomy	40.7	24.87-56.47		
	Pneumonectomy	22.1	12.44-31.82		
Maximum diameter of tumor^*^				2.887	0.089
	< 4 cm	42.7	22.56-62.90		
	≥4 cm	31.6	10.65-52.55		
Number of involved mediastinal lymph nodes^**^				1.428	0.232
	< 3	42.7	21.78-63.68		
	≥3	33.8	11.38-56.28		
Positive subcarinal lymph node^##^				0.400	0.527
	No	44.0	18.63-69.43		
	Yes	33.8	22.32-45.34		
#: Have one case lost in this factor analysis. ^*^: Have five cases lost in this factor analysis. ^**^: Have nine cases lost in this factor analysis. ^##^: Have nine cases lost in this factor analysis.					

**2:**
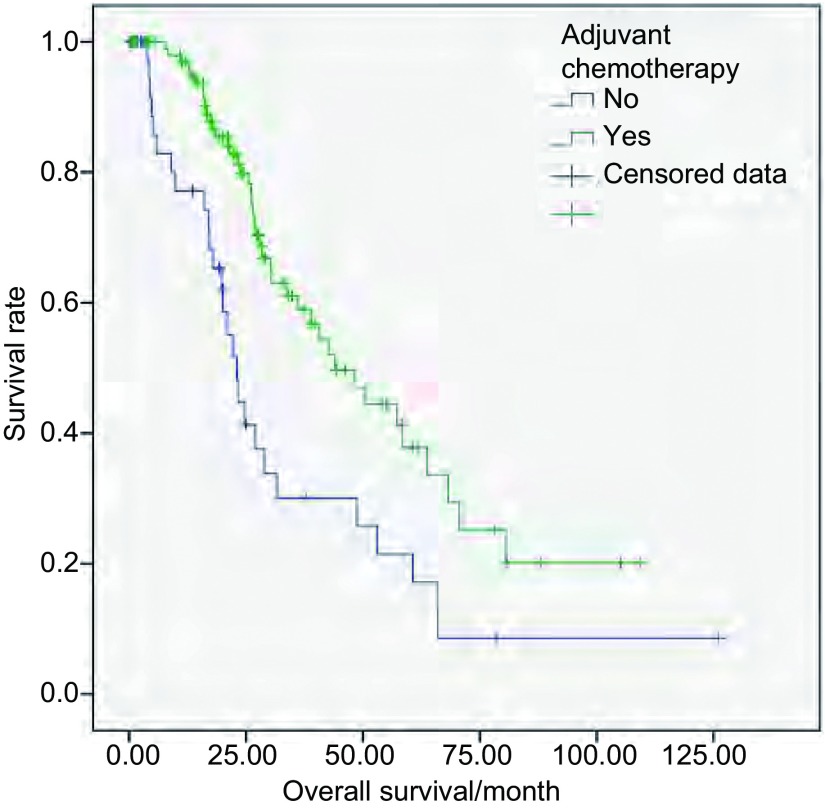
Ⅲa期手术者术后有无辅助化疗的OS曲线 Overall survival (OS) curves of stage Ⅲa surgical patients with or without adjuvant chemotherapy

#### 2.4.2 PFS

单因素分析显示肿瘤家族史和肿瘤最大径对Ⅲa期手术患者PFS的影响有统计学意义（表 3）。其中肿瘤最大径 < 4 cm者与≥4 cm者相比，PFS相差8.6个月。多因素分析显示肿瘤家族史对PFS的影响有统计学意义，有肿瘤家族史者与无肿瘤家族史者相比进展风险增加了69.1%（*P*=0.039, HR=1.691, 95%CI: 1.026-2.787）。肿瘤最大径 < 4 cm与≥4 cm者的PFS曲线见图 3。Ⅲa期中T1-3N2M0期术后放疗与未放疗者的中位PFS分别为21.8个月和13.0个月。两者PFS的差异无统计学意义（χ^2^=2.890, *P*=0.089）。

**3:** 151例患者无进展生存期的单因素分析s Univariate analysis of progresion free survival (PFS) of 151 patients

Factor		PFS/month	95%CI	*χ*^2^	*P*
Gender				2.930	0.087
	Male	14.0	8.68-19.32		
	Female	23.1	19.04-27.10		
Age (yr)				0.001	0.977
	< 60	18.2	11.12-25.22		
	≥60	19.6	14.25-25.01		
Smoke				0.088	0.767
	No	19.3	13.95-24.71		
	Yes	16.5	10.40-22.60		
History of tumor				0.105	0.746
	No	19.2	14.64-23.70		
	Yes	12.7	2.92-22.48		
Family history of tumor				4.361	0.037
	No	19.3	14.88-23.79		
	Yes	16.5	4.44-28.56		
Performance status				0.874	0.350
	0	19.2	14.77-23.57		
	1	19.6	12.29-26.97		
Stage Ⅲa				0.023	0.880
	T4N0/T3-4N1	14.9	8.94-20.86		
	T1-3N2	19.8	14.78-24.88		
Histology				0.393	0.531
	Squamous	16.5	10.05-22.95		
	Non-squamous	19.8	15.05-24.61		
Differentiation				2.101	0.552
	Poor	14.9	8.42-21.38		
	Mediate	19.3	12.27-26.39		
	High	20.1	14.40-25.80		
	Unknown	15.8	0.87-30.79		
Adjuvant chemotherapy				2.399	0.121
	None	14.2	5.96-22.44		
	Yes	19.6	14.56-24.70		
Adjuvant radiotherapy				3.040	0.081
	None	14.0	9.31-18.69		
	Yes	20.0	16.78-23.29		
Style of surgery^#^				0.247	0.619
	Lobectomy	19.3	14.36-24.30		
	Pneumonectomy	14.9	12.39-17.41		
Maximum diameter of tumor^*^				5.628	0.018
	< 4 cm	21.8	17.74-25.92		
	≥4 cm	13.2	9.57-16.83		
Number of involved mediastinal lymph nodes^**^				0.699	0.403
	< 3	19.3	14.47-24.19		
	≥3	16.0	7.55-24.46		
Positive subcarinal lymph node^##^				0.159	0.690
	No	16.0	11.13-20.87		
	Yes	21.3	13.83-28.83		
#: Have one case lost in this factor analysis. ^*^: Have five cases lost in this factor analysis. ^**^: Have nine cases lost in this factor analysis. ^##^: Have nine cases lost in this factor analysis.					

**3:**
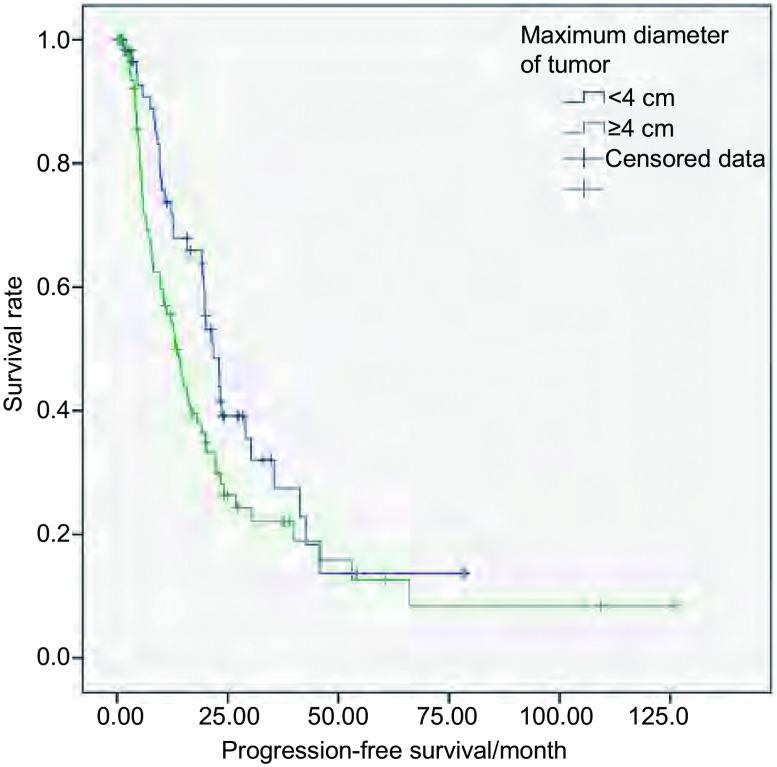
Ⅲa期手术者肿瘤最大径 < 4 cm和≥4 cm的PFS曲线 Progression-free survival (PFS) curves of stage Ⅲa surgical patients with maximum diameter of tumor < 4 cm or ≥4 cm

## 3 讨论

手术虽然是Ⅰ期或Ⅱ期NSCLC患者的标准治疗方式，但由于NSCLC在早期就有全身播散的倾向，故最初确诊时有50%以上的病例已不适于接受手术治疗。对于可以手术切除的Ⅲa期NSCLC，即使是完全切除，其术后复发及死亡的风险仍然较大。有近30%的患者在术后5年内出现局部复发或区域淋巴结转移^[1]^。国内一项回顾性研究^[2]^提示Ⅲ期-N2的NSCLC患者术后的中位OS为22个月。而一项国外研究^[3]^报道Ⅲ期NSCLC手术患者的中位OS为31个月。本研究中151例Ⅲa期NSCLC手术患者的中位OS为38.9个月，中位PFS为19.2个月，与国外研究报道相仿。Ⅲa期中的T4N0/T3-4N1M0期和T1-3N2M0期的中位OS分别为48.7个月和38.9个月，中位PFS分别为14.9个月和19.8个月。两者的OS和PFS均无统计学差异（*P* > 0.05）。与目前2011年国际抗癌联盟修订后的肺癌国际分期Ⅲa期包括T4N0/T3-4N1M0、T1-3N2M0期相符。

目前，由于Ⅲa期NSCLC患者单纯手术后较高的复发率和死亡率，国内外普遍倡导进行术后辅助治疗。术后化疗的作用主要是杀死已经发生的远处微小亚临床转移灶，或者减少和预防发生远处转移。其疗效已得到较多临床研究的证实，目前已成为Ia期以上NSCLC患者术后的标准治疗方式。多次随机对照研究提示对于Ⅲa期NSCLC患者，术后进行以铂类为基础的两药联合化疗的生存期优于未化疗的患者（*P*=0.015），同时Ⅲa-N2期NSCLC患者术后的辅助化疗方案和化疗疗程数对其预后有明显影响^[4]^。Le Chevalier^[5]^、Douiilard^[6]^等的研究结论认为Ⅲa期患者经过术后辅助化疗后生存时间得以延长。潘泓等^[7]^也曾报道Ⅲ期NSCLC切除术后予顺铂为主的化疗，可改善长期生存，5年生存率为27.6%。本研究中单因素和多因素分析均证实了术后辅助化疗的重要性，术后辅助化疗者与未进行辅助化疗者相比，中位OS延长了21个月。所以，对于Ⅲa期手术的NSCLC患者，术后应该进行辅助化疗。

术后放疗作为一种局部治疗方式，可以杀死残留肿瘤及局部亚临床病灶，从而降低肿瘤复发率。所以术后放疗理论上是可以改善治疗效果的。但其临床意义尚有很多争议。目前认为Ⅲ期-N2的NSCLC患者需要有计划的进行术后辅助放化疗。国外一项1998年发表随后又被最近的一些研究更新过的*meta*分析^[8, 9]^统计了11项前瞻性研究中的2, 343例Ⅰ期-Ⅲ期NSCLC患者，结果表明术后放疗对患者的生存期有负性作用，但亚组分析显示术后放疗对生存期的负性作用似乎仅局限于Ⅰ期-Ⅱ期的NSCLC，N2淋巴结转移的患者进行术后放疗是获益的。最近又有几项研究^[6, 10, 11]^证实术后放疗对于手术完全切除后的N2淋巴结转移的NSCLC患者可以延长其生存期。但Wisnivesky等^[12]^认为术后放疗对N2淋巴结转移的老年NSCLC患者的生存期无改善。也有研究^[13]^指出，对于Ⅲa-N2期NSCLC患者，虽然术后放疗不能明显改善患者的5年生存率，但是亚组分析表明有多站N2淋巴结转移者进行术后辅助放疗的PFS与单站N2淋巴结转移者相比具有统计学差异。回顾国内文献，张连民等^[4]^报道术后辅助放疗可以延长Ⅲa-N2期患者的生存期，在腺癌患者中这一趋势最为明显。这与黄国俊等^[14]^的报道一致。也有大量研究^[4-6]^及*meta*分析显示术后放疗可以提高肿瘤的局部控制率，但并不增加或减少Ⅲ期NSCLC的死亡风险。本研究结果显示，不管是对于Ⅲa期总体还是Ⅲa-N2期NSCLC患者，术后辅助放疗对生存期均无明显改善作用。故术后辅助放疗的价值仍有待进一步的研究证实。

## References

[1] Veeramachaneni NK, Feins RH, Stephenson BJK (2012). Management of stage ⅢA non-small cell lung cancer by thoracic surgeons in North America. Ann Thorac Surg.

[2] Ma Q, Liu D, Guo Y (2010). Surgical therapeutic strategy for non-small cell lung cancer with mediastinal lymph node metastasis (N2). Zhongguo Fei Ai Za Zhi.

[3] Steger V, Spengler W, Hetzel J (2012). Pneumonectomy: calculable or non-tolerable risk factor in trimodal therapy for stage Ⅲ non-small-cell lung cancer?. Eur J Cardiothorac Surg.

[4] Zhang LM, Liu XZ, Zhang ZF (2010). A clinical and prognostic retrospective analysis of Ⅲa-N2 non-small cell lung cancer. Zhonghua Wai Ke Za Zhi.

[5] Le Chevalier T, Arriagada R, Le Pechoux C (2004). Cisplatin-based adjuvant chemotherapy in patients with completely resected non-small-cell lung cancer. New Eng J Med.

[6] Douillard JY, Rosell R, De Lena M (2008). Impact of postoperative radiation therapy on survival in patients with complete resection and stage Ⅰ, Ⅱ, or ⅢA non-small-cell lung cancer treated with adjuvant chemotherapy: The Adjuvant Navelbine International Trialist Association (ANITA) randomized trial. Int J Radiat Oncol Biol Phys.

[7] Pan H LD, Mao NQ (2001). Clinical observation of adjuvant chemotherapy after radical surgery for stage Ⅲ non-small cell lung cancer. Chin Clin Oncol.

[b8] 8Postoperative radiotherapy in non-small-cell lung cancer: systematic review and meta-analysis of individual patient data from nine randomised controlled trials. PORT *Meta*-analysis Trialists Group. Lancet, 1998, 352(9124): 257-263.

[9] Stewart L, Arriagada R, Brichet AH (2008). Postoperative radiotherapy for non-small cell lung cancer. Semin Thorac Cardiovasc Surg.

[10] Morgensztern D, Waqar S, Subramanian J (2012). Prognostic significance of tumor size in patients with stage Ⅲ non-small-cell lung cancer: a surveillance, epidemiology, and end results (SEER) survey from 1998 to 2003. J Thorac Oncol.

[11] Gao YS, Xing XZ, Shao K (2008). Analysis of prognostic factors in 1826 patients with completely resected non-small cell lung cancer. Zhonghua Zhong Liu Za Zhi.

[12] Wisnivesky JP, Halm EA, Bonomi M (2012). Postoperative radiotherapy for elderly patients with stage Ⅲ lung cancer. Cancer.

[13] Matsuguma H, Nakahara R, Ishikawa Y (2008). Postoperative radiotherapy for patients with completely resected pathological stage ⅢA-N2 non-small cell lung cancer: focusing on an effect of the number of mediastinal lymph node stations involved. Int Cardiovasc Thorac Surg.

[14] Huang GJ, Fang DK, Cheng GY (2006). Surgical therapeutic strategy for non-small cell lung cancer with (N2) mediastinal lymph node metastasis. Zhong Hua Zhong Liu Za Zhi.

